# A Rare and Complex Case of Non-suicidal Self-Mutilation in a Patient With Schizophrenia

**DOI:** 10.7759/cureus.33269

**Published:** 2023-01-02

**Authors:** Ashna Khanna, Davin Agustines

**Affiliations:** 1 College of Osteopathic Medicine of the Pacific, Western University of Health Sciences, Pomona, USA; 2 Psychiatry, Olive View University of California Los Angeles Medical Center, Los Angeles, USA

**Keywords:** poverty, drug and substance abuse, command auditory hallucinations, digital gangrene, major self-mutilation, auto-amputation, schizophrenia and other psychotic disorders

## Abstract

The literature describing acts of non-suicidal self-mutilation (NSSM) in the adult population is limited. Of the cases that document NSSM, a disproportionate number of these individuals have a history of psychiatric illnesses. Although the motivation to perform NSSM varies across patients, the literature suggests that past self-injurious behaviors, extreme religious delusions, and command hallucinations are the most significant risk factors. The primary forms of NSSM include ocular, genital, and limb mutilation. Limb mutilation is the least common of the three and typically occurs proximal to the wrist or hand. Here, we present a rare case involving a 42-year-old man with schizophrenia who was hospitalized due to osteomyelitis of his autoamputated digits. This case is unique in involving multiple digits of the hand and using a rare amputation method. We aim to compare this case with the existing body of work on NSSM and identify factors that may predispose patients to act on these extreme impulses. We also highlight a novel interventional program that reduces psychiatric and medical comorbidities.

## Introduction

Schizophrenia is a rare but severe mental illness that affects approximately 1% of the general population [1]. It is diagnosed based on positive symptoms such as delusions and hallucinations, cognitive symptoms such as disorganized thinking and speech, and negative symptoms such as apathy, anhedonia, and catatonia [1].

Non-suicidal self-mutilation (NSSM) is the deliberate alteration or damage to one’s body without conscious suicidal intent or sexual arousal [2,3]. In the context of psychiatric illness, NSSM is further divided into major and minor (or superficial) acts of self-mutilation. Minor acts of self-mutilation are more common and include nail-biting, hair-pulling, and skin-cutting [3,4]. In contrast, major acts of self-mutilation are more often sporadic and result in permanent organ damage [3]. Examples of major self-mutilation include enucleation of the eye, genital self-mutilation, and autoamputation of the limbs [3]. Of the three, limb amputation consists of less than 10% of acts of major self-mutilation [5,6]. While minor acts of NSSM are commonly associated with obsessive-compulsive disorder (OCD), autism spectrum disorder, or borderline personality disorder, major acts of self-mutilation are more often associated with schizophrenia and substance abuse [4,7]. A study examining the literature for major acts of self-mutilation found that psychotic illness, most commonly psychosis secondary to schizophrenia spectrum disorders, was the most common diagnosis among 189 cases of NSSM [7]. Additionally, of those diagnosed with psychotic illness, most individuals were off antipsychotic medication at the time of NSSM [6,7]. Among patients with schizophrenia or frank psychosis, predictors of self-mutilation included past self-injurious behaviors, religious delusions, and command hallucinations [2,5].

Providing consistent care for patients with severe mental illness can be incredibly challenging due to a number of barriers and limitations to care [8]. Individuals with severe mental illness often experience multiple hospitalizations and worsening psychiatric and medical comorbidities. Assertive Community Treatment (ACT) is a longstanding treatment model utilizing frequent check-ins, low participant-staff ratios, and community outreach to help people who have severe mental illness [9]. In California, a modification of the ACT treatment model was developed after the passage of the Mental Health Services Act in 2004, which taxed 1% on adjusted gross incomes in excess of $1 million to help fund community-focused outreach care. The Full-Service Partnership (FSP) program is a core component that developed as a result. FSP involves an intensive outpatient case management team that identifies patients with severe mental illness and provides them with treatment (counseling and pharmacotherapy) and other supportive services to assist with employment, housing, and education [10]. The primary difference between ACT and FSP is the wraparound support, including flexible spending capability to provide basic needs (food, clothing, temporary shelter, and transportation services) to FSP participants. In addition, FSP programs allow for a more individualized and accessible approach to help patients with severe mental illness maintain their medical and psychiatric appointments. The hope is that participating in an FSP program makes individuals less likely to require emergency department visits and hospitalizations. Data from a study that examined the impact of FSP programs showed that emergency department visits decreased by more than half among FSP participants [10].

To determine the rarity of this case of NSSM, we searched PubMed for studies published in English describing the occurrence of self-mutilation in psychosis or psychiatric illness using subject headings, keywords, abstracts, and titles from 1960 to 2020. The search terms used to conduct this search included (autoamputation OR self-mutilation) AND (psychosis OR psychiatric illness OR schizophrenia). The search results were further filtered to include autoamputation of the limbs. In addition, we excluded acts of self-mutilation associated with suicidal ideation or sexual arousal. Minor acts of NSSM, such as skin cutting and nail-biting, were also excluded. Among the remaining cases, we identified only one case of repeated digital autoamputation in a patient with OCD [11].

We present a rare and complex case involving Mr. R, a 42-year-old man with schizophrenia, who was brought in by his FSP team after he was found repeatedly constricting the circulation of his digits. Ultimately, this resulted in the autoamputation of his digits. We review this case within the existing literature on NSSM and identify risk factors and predictors of self-mutilation. We also discuss the opportunities FSP programs offer in treatment and intervention.

## Case presentation

Mr. R is a 42-year-old Hispanic male diagnosed with schizophrenia, complicated by decades of substance misuse and chronic homelessness. Mr. R was first admitted to medical service at our facility in October 2021 due to his left second-digit autoamputation. When the outreach FSP team first encountered Mr. R, he had constricted his left second digit with a rubber band. Unfortunately, this resulted in the development of dry gangrene, and the digit ultimately autoamputated prior to medical intervention. Fortunately, the FSP team convinced him to go to the hospital, where he received wound care and psychiatric treatment.

Following his wound care, Mr. R was transferred to the inpatient psychiatric unit due to the acute decompensation of his schizophrenia. At this time, he demonstrated auditory command hallucinations, disorganized thinking and speech, and indifference toward his situation. According to the patient, he had been seen at another facility two months prior for a similar episode involving his right thumb. When Mr. R was hospitalized, he requested to be started on oral haloperidol, as he felt this medication had best controlled his auditory hallucinations. However, he later transitioned to haloperidol decanoate 150 mg intramuscularly every four weeks due to his history of medication non-compliance. Once Mr. R’s auditory hallucinations lessened, and he stopped endorsing command auditory hallucinations to wrap bands around his fingers, he was discharged to an assisted living facility. During this hospitalization, he demonstrated some improvements in the linearity of thinking and cognitive processing. However, he did not gain insight into the permanent physical damage he had inflicted upon himself.

Shortly after discharge, Mr. R was brought in by his FSP team again after he tied rubber bands around his distal left middle digit, resulting in dry gangrene and autoamputation. Mr. R was agitated and violent toward hospital staff, requiring restraints and emergent intramuscular medication. When asked why he amputated his finger, the patient stated, “of course, I had to,” and described experiencing command hallucinations, again telling him to wrap bands around his fingers. His left middle finger, after autoamputation, was sent to pathology. It measured 5 cm in length, 2 cm dorsal to palmar/volar aspect, and 2 cm left to right. The nail was also black and necrotic, measuring 1.7 × 1.6 cm. The serially sectioned specimen revealed black, gangrenous bone and soft-tissue necrosis. The wound culture returned positive for diphtheroid and rare enterococcus species. Upon consultation with Infectious Disease, Mr. R was placed on amoxicillin/clavulanic acid 875 mg/125 mg and minocycline 100 mg to treat his left middle finger osteomyelitis.

Mr. R was again found to have positive, cognitive, and negative symptoms of schizophrenia and a positive urine drug screen for amphetamines. While in the hospital, Mr. R could not verbalize a self-care plan or explain why he continued to amputate his fingers. He continued to lack the insight and judgment required to grasp the severity of his psychiatric illness and the permanent physical damage he was inflicting upon himself. Mr. R denied any suicidal or homicidal ideation but continued to endorse auditory and visual hallucinations. While he would not describe the visual hallucinations he experienced, he did describe his auditory hallucinations as a single voice, sometimes multiple voices, all commanding him to wrap rubber bands around his fingers. These voices increased substantially in intensity prior to hospitalization. Mr. R had a significant history of substance misuse and addiction in the past. However, during this visit, his addiction fit the criteria for mild classification as he denied drug seeking but admitted to accepting amphetamines and alcohol if offered to him by acquaintances. He continued to receive haloperidol decanoate 150 mg intramuscularly every four weeks for his psychotic symptoms.

Over time, the linearity of his thinking and cognitive processing, which was initially highly disorganized, improved. Mr. R’s speech was terse, and at times he was found responding to talking to himself in his room. However, he remained oriented to person, place, and time. For most of Mr. R’s hospitalization, he displayed constricted affect and congruent mood but had a steady lessening of his auditory hallucinations and ceased to experience the auditory command hallucinations. In addition, he became more interactive with the staff at the hospital.

Unfortunately, two weeks into Mr. R’s hospital stay, he contracted coronavirus disease 2019 (COVID-19) and was placed in isolation with other COVID-19-positive patients for 14 days. While in isolation, Mr. R became withdrawn but asked to meet with his FSP team as his medical condition improved. When the FSP team spoke with Mr. R at his bedside, he demonstrated an understanding of why he needed to be compliant with medications and the importance of developing social circles away from other substance-using individuals. In addition, his FSP team helped him develop coping and fallback strategies to help him avoid rehospitalization, for which Mr. R was appreciative. With his FSP team’s intervention and ongoing intensive psychiatric treatment, Mr. R developed an appreciation of the importance of remaining involved in mental health treatment. Ultimately, he was accepted into another assisted living environment, this time with intensive mental health wraparound services to maintain medical compliance and monitor for return of NSSM. To our knowledge, he has not engaged in NSSM since this discharge.

## Discussion

This case is complicated on many levels. For one, Mr. R has experienced chronic homelessness for over 20 years, making consistent management and follow-up of his schizophrenia incredibly difficult. He also struggled with decades of substance misuse and addiction, with the onset of use in his teenage years leading to longstanding issues with the criminal justice system. At the time of this current hospitalization, Mr. R’s substance use disorder fit the criteria for mild classification. While Mr. R no longer actively sought out drugs of abuse, he would accept them when offered. He had no clear understanding at the time of his hospitalizations about the role of substance use in worsening his psychotic symptoms. In addition, he lacked any social support and had strained family relations from a lifetime of severe mental illness. These social and economic factors further contributed to the cyclical course of Mr. R’s illness. Nevertheless, while these challenges are not unique to Mr. R, his presentation of NSSM is highly uncommon. As mentioned previously, a PubMed search identified only one case of repetitive digit autoamputation [11].

While Mr. R did not demonstrate religious delusions, he admitted to experiencing command auditory hallucinations and, with subsequent hospitalizations, evidence of prior self-injury. These factors have been associated with an increased risk of NSSM in individuals with schizophrenia [2,5]. Additionally, Mr. R’s inability to maintain consistent follow-up for antipsychotic treatment has also been identified as a risk factor for NSSM [5].

Mr. R had access to homeless outreach programs; however, the extent of his mental illness and addiction necessitated a significant level of support. Fortunately, because of his FSP team’s ability to go into the field, provide voluntary transport, and engage in intensive psychotherapeutic endeavors both in the field and at the hospital, Mr. R received medical and psychiatric attention before his self-inflicted injuries became critical.

This case demonstrates how FSP programs can support access to mental health care within underserved communities [10]. Furthermore, data from studies on FSPs demonstrate that the length of participation in the program correlates with a significant reduction in emergency department visits over time [10]. Based on this, we postulate that with continued participation in the program and community-based support, Mr. R’s risk of subsequent NSSM will decrease. This case, and others describing NSSM, highlights the need for consistent and reliable treatment of NSSM. For example, several papers demonstrate that early and ongoing intervention and treatment of schizophrenia reduces the risk of NSSM [5-7]. Although it is difficult to predict who will go on to enact NSSM, individuals who perform NSSM tend to be those with untreated psychotic illness [5]. Thus, early identification and consistent treatment for individuals with schizophrenia spectrum disorders may reduce the incidence of NSSM.

## Conclusions

The symptoms of schizophrenia can be incredibly disabling and, in extreme cases, as demonstrated here, can result in self-inflicted disability. While there have been previous accounts of NSSM associated with psychosis, serially repeated ligature has not been well-documented. The degree of NSSM in this patient likely reflects the challenges associated with managing mental illness in the setting of extreme poverty and addiction. This case report and others of similar nature highlight the cyclical effects of poverty and addiction that contribute to and continually exacerbate mental illness.
